# Pyogenic Spondylodiscitis due to *Streptococcus constellatus* in an Immunocompromised Male Patient: A Case Report and Review of the Literature

**DOI:** 10.1155/2019/9364951

**Published:** 2019-11-15

**Authors:** Charalampos Potsios, Panagiota Xaplanteri, Vasileios Zoitopoulos, Panagiotis Patrinos, Iro Ioanna Giannakopoulou, Ilektra Tzivaki, Theodora Kafentzi, Konstantina Samanta, Konstantina Filioti, Panagiotis Sideris, Konstantinos Letsas, Konstantinos Koukios

**Affiliations:** ^1^Department of Internal Medicine, General Hospital of Eastern Achaia, Aigio, Greece; ^2^Department of Microbiology, University General Hospital of Patras, Patras, Greece; ^3^Department of Microbiology, General Hospital of Eastern Achaia, Aigio, Greece

## Abstract

Pyogenic spondylodiscitis is a primary infection of the intervertebral disc and is a rare entity. Here, we describe the case of a 64-year-old male patient, a professional breeder, who attended the Emergency Department with sciatica and back pain that was worsening for a week. The patient had no history of surgery or trauma. The patient had poor oral hygiene. Magnetic resonance imaging (MRI) scan showed lumbar spondylodiscitis, and blood cultures revealed *Streptococcus constellatus*. The patient was initially treated with vancomycin but due to renal failure deterioration, the treatment was changed to daptomycin for 8 weeks. During hospitalization, he endured renal injury and nosocomial respiratory tract infection. The patient was discharged with no further complications. Follow-up revealed improvement of neurological signs. In our case, it seems that poor oral hygiene was the cause of bacteremia, which underlies the importance of a good oral health status in immunocompromised patients not only to prevent but also to successfully eliminate any dental source of infection. *S. constellatus* is an extremely rare pathogen and to our knowledge only two other cases of pyogenic spondylodiscitis are reported in the literature. Early diagnosis is very important for the prognosis of these patients.

## 1. Introduction

Spondylodiscitis is a primary infection of the intervertebral disc by a pathogen and is a rare infection of the musculoskeletal system [1, 2]. The symptoms are not specific, the diagnosis is delayed, and the source of the infection is not obvious. At the time of diagnosis, there is also evident secondary infection of neighboring vertebral bodies, shown via conventional X-ray, and the source of the culprit pathogen is no longer easy to determine. As a result, both primary and secondary infections are used to describe this rare entity [2]. *Streptococcus constellatus* belongs to the *S. anginosus group* and is a very rare cause of pyogenic spondylodiscitis [1]. Here, we describe the case of a 64-year-old male immunocompromised patient, a professional breeder, who attended the Emergency Department with sciatica and back pain that was worsening for a week. Magnetic resonance imaging (MRI) scan showed lumbar spondylodiscitis, and blood cultures revealed *S. constellatus*.

## 2. Case Presentation

A 64-year-old male patient, a breeder, with a history significant for type II diabetes mellitus, chronic obstructive pulmonary disease, arterial hypertension, and severe alcohol abuse attended the Emergency Department complaining about sciatica and gradually worsening back pain that commenced a week before. The patient had no history of surgery or trauma but had poor oral hygiene.

Palpation revealed tenderness over the lower lumbar region. No spinal deformities, and no motor or sensory deficits were present. On admission, temperature was 38°C, blood pressure 90/60 mmHg, pulse rate 105 beats/min, and oxygen saturation 93% (FiO_2_: 21%).

The initial laboratory findings showed elevated white blood cell count 13100/mm^3^ (normal value 5000 to 10000), 81% neutrophils, thrombocytopenia with platelet count 49000/mm^3^ (normal value 150000–400000/mm^3^), raised levels of C-reactive protein (CRP) 38.2 U/L (normal value <0.33 U/L). Ultrasound examination of the abdomen revealed small paraaortic and iliac lymph nodes with size up to 9 mm. Computed tomography (CT) and subsequent magnetic resonance imaging (MRI) scan were consistent with spondylodiscitis of the lumbar level L4-L5. Inflammation also extended to the spinal tube with pathological vascularization of the mandibula in the anterior epidural space at the L4-L5 level, creating pressure in the spinal canal. A large central herniated disc of at least 6 mm in size was present at the L4-L5 level exercising significant pressure on the spinal cord. Due to his profession and the high incidence of brucellosis in the area where he lived, brucellosis was firstly suspected. Blood samples were inoculated into BAC/TAlert® 3D (bio-Merieux) blood aerobic and anaerobic culture bottles. Needle biopsy was not performed. Infective endocarditis was ruled out by transthoracic ultrasound.

Identification of the culpable microorganism was performed by BBL™ Crystal™ Identification Systems for Gram-positive bacteria (BD Diagnostics, Le Pont de Claix, France). Antibiotic susceptibility testing was performed by the disk diffusion method for ampicillin, ceftriaxone, chloramphenicol, penicillin, vancomycin, teicoplanin, clindamycin, and erythromycin, according to the European Committee on Antimicrobial Susceptibility Testing guidelines [3].

From the blood cultures, *Streptococcus constellatus* was isolated, which was sensitive to ampicillin, ceftriaxone, chloramphenicol, penicillin, vancomycin, and teicoplanin and resistant to clindamycin and erythromycin.

Due to reported severe allergy to *β*-lactams, the patient was initially treated with vancomycin 1 g twice a day. Because of deterioration of renal failure, the treatment changed to daptomycin since it is also a lipopeptide antibiotic, with good diffusion into the discs, and effective against Gram-positive organisms. Therefore, daptomycin was considered the best choice for the patient, although not included in the antimicrobial susceptibility testing. The patient obtained intravenous antibiotic therapy for a total of 8 weeks. During hospitalization, he endured renal injury and nosocomial respiratory tract infection. The patient was discharged with no further complications. Follow-up revealed improvement of neurological signs.

## 3. Ethical Approval

The study was approved by the Scientific Council of General Hospital of East Achaia (Approval Number 23) and waived the need for informed consent, since isolates were recovered under routine diagnostic procedures.

## 4. Discussion

Spondylodiscitis is due to bacteria (pyogenic), tuberculosis or fungi (granulomatous infection), or parasites [4]. Pyogenic spondylodiscitis is a very rare entity. Its incidence in developed countries is estimated to be 4 to 24 per million per year, with a prevalence in male elderly patients [1]. The incidence is increasing due to improvement of diagnostic methods [5]. Hospital mortality varies from 2% to 17% [2].

In pyogenic spondylodiscitis, the main source of infection is haematogenous spread from a distant focus that in the descending order is the genitourinary tract (17%), the skin and soft tissues (11%), intravascular devices (5%), the gastrointestinal tract (5%), the respiratory tract (2%), and the oral cavity (2%) [1]. In this case, it is called endogenous spondylodiscitis and the culprit microorganism can be blood-borne both by arteries and veins [2]. In our case, the bad oral hygiene of the patient proved to be the source of bacteremia and subsequent spinal infection.

Risk factors include diabetes mellitus, advanced age, intravenous drug abuse, immunosuppression, multimorbidity, rheumatic diseases, renal and liver failure, cardiovascular diseases, obesity, HIV, and previous spinal surgery [1, 2]. Our patient suffered from diabetes mellitus and alcohol abuse, therefore was considered immunocompromised.

Differential diagnosis includes erosive osteochondrosis, pathological fracture, polymyalgia rheumatica, vertebral hemangioma, osteoporosis, cancer-related destruction, Scheuermann's disease, and ankylosing spondyloarthritis [2, 6]. The infection is mostly monomicrobial. *Staphylococcus aureus* is the main culprit for almost half the nontuberculosis cases for community-acquired haematogenous vertebral osteomyelitis if patients over 50 years old and intravenous drug users [1]. Enterobacteriaceae follow and in these cases the pathogen's source is the urinary tract [1, 7]. Coagulase-negative *staphylococci* (*CoNS*) are responsible for 5%–16% of cases. *Staphylococcus epidermidis* is the most frequently identified species in postoperative bacteremia or related to intracardiac devices [1]. Anaerobes represent less than 4% of cases and are related to postoperative discitis, and contiguous spread [1]. Brucellosis is responsible for 7.5%–30% of spondylitis, mainly due to *Brucella melitensis* [1].

*Streptococci* and *enterococci* are responsible for 5%–20% of pyogenic spondylodiscitis cases [1, 8]. To our knowledge, *Streptococcus constellatus* is an extremely rare pathogen and only two other cases are reported in the literature involving an elderly man of 72 years and a case of a 14-year-old male teenager [4, 9]. *S. constellatus* is a group C Gram-positive *Streptococci*, which form colonies with *β*-haemolysis and belongs to the *S. anginosus group* together with *S. anginosus* and *S. intermedius* [3]. It is part of normal flora of the oral cavity, gastrointestinal, and urogenital system [4]. It requires microaerophilic environment which means that it can survive in parts of the human body that are oxygen-depleted such as the spinal disk [9].

The symptoms of pyogenic spondylodiscitis are nonspecific [1]. Therefore, diagnosis is delayed and has been reported to be two to six months [2]. During primary medical care, the patients are frequently thought to be suffering from degenerative diseases of the spinal column [1, 2]. They are then treated correspondingly, even though the prognosis is better with early diagnosis. Pain may be absent in 15% of patients, but if it exists it involves the back or the neck, is constant, and worsens at night. It may radiate to the chest or abdomen misleading diagnosis [1]. Fever is present in less than 50% of cases [1]. One-third of patients have neurological deficits related to epidural abscess and delayed diagnosis. These deficits include sensory deficit, leg weakness or paralysis, and sphincter loss [1]. On examination, spinal tenderness is present in 78–97% of patients [1]. Laboratory findings include elevated erythrocyte sedimentation rate (ESR) and C-reactive protein (CRP) in over 90% of patients, but they lack specificity [1]. The leucocyte count is normal in most immunocompromised or elderly patients and seems to be high only in one-third of cases. On the other hand, 70% of patients have anemia and 50% have elevated serum alkaline phosphatase [1]. Our patient's physical examination revealed tenderness over the lower lumbar region and had a temperature of 38°C, elevated white blood cell count, thrombocytopenia, and raised levels of C-reactive protein.

As most infections are monomicrobial and blood-borne, blood culture is the best way to identify the culprit microorganism. Blood cultures are positive in 70% of cases, given that the patients were not previously treated with antibiotics, and at least two or three pairs of blood cultures are obtained [1, 2]. Biopsy cultures in adult patients are appropriate for cases with negative blood cultures. Biopsy should be repeated if the first biopsy culture proves negative, whereas bone marrow culture should be performed if brucellosis is suspected [1]. In cases of prior antibiotic usage, negative cultures after 48 h incubation, or the presence of fastidious microorganisms, molecular diagnostic methods are very useful [1, 6]. Those methods include broad-range 16S rDNA and species-specific polymerase chain reaction (PCR) targeting *S. aureus* and MRSA strains by amplification of the *mecA* gene [1, 6]. In our case, the culprit microorganism was isolated from blood culture; therefore, biopsy was not performed.

Histology is helpful to differentiate pyogenic and granulomatous disease and should be supplementary to cultures [1]. Radiological methods and imaging help establish the diagnosis, but they appear late usually 2–8 weeks after onset of symptoms. Plain radiography may reveal early lesions such as loss of disc height and later ones such as paravertebral soft tissue mass [1]. Technetium-99m-methylene diphosphonate bone scintigraphy has a specificity of 78%, leading to false-positive results and should be combined with Gallium-67 scintigraphy [1]. Fluorine-18 ﬂuorodeoxyglucose (F-18 FDG) positron emission tomography (FDG-PET) can distinguish infection from degenerative changes better than magnetic resonance imaging (MRI) as the uptake of F-18 FDG is proportionally linked to the enhancement of glucose metabolism in the inflammatory cells [1, 2]. The disadvantage of this method is that there can be no distinction from malignancies [2]. MRI reveals best the sites of nerve or spinal cord compression and abscess formation but is of little value regarding follow-up of patients [10].

Computed tomography (CT) is most useful in lesion provoked by tuberculosis but is inferior to MRI as far as abscesses are concerned. It is mostly used as guidance of spinal biopsy [1, 10]. The imaging method of choice for the diagnosis of spondylodiscitis is MRI with gadolinium, with 96% sensitivity and 93% specificity, simultaneously providing information for the epidural space and spinal cord [1]. In our patient, diagnosis was set by MRI, which revealed the exact site of infection and the pressure on the spinal canal.

Treatment focuses on the elimination of infection and pain, and the preservation of function and structure of the spine. In this direction, parenterally administered antimicrobial therapy combined with immobilization for at least six weeks are appropriate [1, 2]. Immobilization is required if there is risk of spinal instability and pseudoarthroses (16% to 50%) [1, 2]. The use of reclining ortheses decreases the stress of the infected area [2]. The duration of antimicrobial therapy varies in many studies, and its reported length range is from 3 to 12 weeks [1, 2]. Oral antibiotic therapy for a period of six weeks to three months is recommended for nonspecific spondylodiscitis. Researches in animals show that clindamycin, aminoglycosides, and glycopeptides penetrate and diffuse well into healthy discs, but this observation should be further elucidated in case of infection [1]. The Infectious Diseases Society of America (IDSA) guidelines define a 6-week therapy for patients with nonspecific spondylodiscitis. For oral therapy, quinolones, clindamycin, and cotrimoxazole can be used. On the other hand, *β*-lactams should be avoided because they have poor bioavailability and bone penetration [6]. In our case, the patient reported allergy to *β*-lactams and in combination with their poor bioavailability and bone penetration, vancomycin was the first choice for treatment. Due to deterioration of the patient's renal function, vancomycin was then changed to daptomycin. Daptomycin is a lipopeptide antibiotic, with good diffusion into the discs and effective against *Streptococci* [3]. Therefore, daptomycin was considered the best choice for the patient, although not included in the antimicrobial susceptibility testing.

Surgical intervention in the form of emergency surgery is the treatment of choice when there is neural compression, extensive bone destruction, severe pain that is difficult to deal with, epidural abscess, or failure of conservative management [1, 2]. In the case of paravertebral and intradiscal abscesses in patients with no neurological deficits, radiologically guided percutaneous drainage offers an alternative to surgery and also helps in the identification of the culprit microorganism [1]. In our patient, the pathogen was isolated from the blood culture, he was treated with antibiotics, and follow-up revealed improvement of neurological signs. Therefore, surgical treatment was avoided.

Complications include disability due to pain or neurological deficits in 30% of patients [1]. Motor deficits in patients who underwent surgery and had preoperative neurological deficits are 30% and hypesthesia 90% [1]. High rates of PCR over 150 mg/L on admission and infection due to *S. aureus* are related to motor deficits. A score of 3 or less in the Rankin scale is related to good functional outcome in patients with spinal cord compressions [5].

The duration of follow-up is not clear, and relapses have been reported even years after the original infection [1, 2]. Prolonged antibiotic therapy has not been associated with better functional outcome or relapses [5]. Till now, our patient's follow-up shows improvement of neurological signs and no relapse has been detected.

Pyogenic spondylodiscitis is a rare entity, the incidence of which is rising, due to the availability of more effective diagnostic tools. It requires a high index of suspicion. Isolation of the culpable microorganism is vital in order to choose the most effective antimicrobial therapy. The optimal treatment duration should be more elucidated as it is a highly heterogeneous disease, which prevents the formulation of treatment recommendations. Antibiotic treatment for 6 weeks appears to be sufficient [6]. Surgical intervention is important for pain alleviation and function restoration [1]. Our patient was treated with antibiotics for a total period of 8 weeks.

In our case, it seems that poor oral hygiene was the cause of bacteremia due to *S. constellatus* and the subsequent spondylodiscitis. A good oral health status is of critical importance especially in immunocompromised patients. In those patients, there should be systematic elimination of all potential oral septic foci. Another point of great importance is early diagnosis of this disease that has nonspecific symptoms and can easily be misdiagnosed with detrimental effects for the outcome.

## References

[1] Gouliouris T., Aliyu S. H., Brown N. M. (2010). Spondylodiscitis: update on diagnosis and management. *Journal of Antimicrobial Chemotherapy*.

[2] Sobottke R., Seifert H., Fätkenheuer G., Schmidt M., Gossmann A., Eysel P. (2008). Current diagnosis and treatment of spondylodiscitis. *Deutsches Aerzteblatt International*.

[3] The European Committee on Antimicrobial Susceptibility Testing (2013). Breakpoint tables for interpretation of MICs and zone diameters. http://www.eucast.org.

[4] Lim S. W., Lim H. Y., Kannaiah T., Zuki Z. (2017). *Streptococcus constellatus* spondylodiscitis in a teenager: a case report. *Malaysian Orthopaedic Journal*.

[5] Lemaignen A., Ghout I., Dinh A. (2017). Duration of Treatment for Spondylodiscitis) study group. Characteristics of and risk factors for severe neurological deficit in patients with pyogenic vertebral osteomyelitis: a case-control study. *Medicine*.

[6] Herren C., Jung N., Pishnamaz M., Breuninger M., Siewe J., Sobottke R. (2017). Spondylodiscitis: diagnosis and treatment options-a systematic review. *Deutsches Aerzteblatt International*.

[7] Zimmerli W. (2010). Vertebral osteomyelitis. *New England Journal of Medicine*.

[8] Seng P., Vernier M., Gay A., Pinelli P.-O., Legré R., Stein A. (2016). Clinical features and outcome of bone and joint infections with streptococcal involvement: 5-year experience of interregional reference centres in the south of France. *New Microbes and New Infections*.

[9] Gangone R., Findlay I., Lakkireddi P. R., Marsh G. (2009). A very rare spontaneous group-C *Streptococcal constellatus* spondylodiscitis: a case report. *Journal of Orthopaedics*.

[10] Riccio S. A., Chu A. K. M., Rabin H. R., Kloiber R. (2015). Fluorodeoxyglucose positron emission tomography/computed tomography interpretation criteria for assessment of antibiotic treatment response in pyogenic spine infection. *Canadian Association of Radiologists Journal*.

